# Conceptual disorganization as a mediating variable between visual learning and metacognition in schizophrenia

**DOI:** 10.3389/fpsyt.2023.1278113

**Published:** 2023-12-20

**Authors:** Cristiana Montemagni, Claudio Brasso, Silvio Bellino, Paola Bozzatello, Vincenzo Villari, Paola Rocca

**Affiliations:** ^1^Dipartimento di Neuroscienze "Rita Levi Montalcini", Università Degli Studi di Torino, Turin, Italy; ^2^Dipartimento di Neuroscienze e Salute Mentale, A.O.U. Città Della Salute e Della Scienza di Torino, Turin, Italy

**Keywords:** path analysis, mediation, visual learning, metacognition, mastery, conceptual disorganization, schizophrenia

## Abstract

**Objectives:**

The aim of this study was to evaluate the relative contributions of visual learning and conceptual disorganization to specific metacognitive domains in a sample of outpatients with stable schizophrenia.

**Methods:**

A total of 92 consecutive outpatients with stable schizophrenia were recruited in a cross-sectional study. We analyzed the data with five path analyses based on multiple regressions to analyze the specific effect of visual learning on metacognitive capacity and metacognitive domains and the possible mediating role of conceptual disorganization.

**Results:**

We found that (i) visual learning was negatively correlated to metacognitive capacity and its domains on the one hand and conceptual disorganization on the other hand; (ii) conceptual disorganization was negatively associated with metacognition and its domains; and (iii) when the mediation effect was considered, conceptual disorganization fully mediated the relationship between visual learning and mastery, whereas it served as a partial mediator of the effect of visual learning on the other metacognition domains, i.e., self-reflectivity, understanding others’ mind, and decentration.

**Conclusion:**

These results delineate an articulated panorama of relations between different dimensions of metacognition, visual learning, and conceptual disorganization. Therefore, studies unable to distinguish between different components of metacognition fail to bring out the possibly varying links between neurocognition, disorganization, and metacognition.

## 1 Introduction

Metacognition (MC) is a complex and multidimensional construct that includes a wide spectrum of processes involving semi-independent abilities or cognitive acts that contain primarily reflexive qualities (1), ranging from the discrete ones, in which an individual identifies a particular emotion or a precise thought, to the more synthetic ones, in which a person integrates separate thoughts and produces holistic representations of oneself or others (2, 3). In doing so, a person is not only passively acquiring information but also building a coherent narrative and developing meaning from their experiences (4–6).

The “integrative model” proposed by Hasson-Ohayon et al. (7) describes MC as a spectrum of activities ranging from the awareness of and reflection upon discrete and specific mental experiences to the ability to grasp reciprocal relationships between thoughts, emotions, and underlying intentions, integrating and synthesizing them into something broader, i.e., a coherent and usable representation of experience and a complex and integrated sense of themselves and others over time and their place in their community rather than fragmented one, in order to find ways to live a more full and satisfying life.

MC impairment has been known in schizophrenia (SZ) for a long time (8), but only recently has MC received major attention in SZ research. The reason for this greater interest stems from the main role of MC in developing a consistent subjective sense of personal identity (9, 10) and interpersonal networks (11–13).

Moreover, there is evidence of conceptual links between MC and other related but independent constructs, such as neurocognition (NC) and social cognition (SC) (14–16), which are more focused on the level of exactness of perceptions and representations, while in contrast, MC focuses on psychological experience synthesis into mental representations with a large variety in terms of complexity, adaptiveness, and flexibility (17).

Furthermore, even if a number of studies suggest that reasonable neurocognitive functioning is a necessary but not sufficient prerequisite to intact SC and MC in SZ (16, 18, 19), the relationships between NC, SC, and MC have not yet been fully elucidated and could be influenced or moderated by additional factors.

Disorganized symptoms, which reflect a characteristic underlying dimension close to the core of the illness, have proven to be a moderator between NC and both SC and MC, given the influence they have on the effectiveness of synthesis of discrete information into an organized whole, a critical factor of both SC and MC (20). The meta-analysis by Arnon-Ribenfield et al. (21) has shown a large inverse relationship between MC and disorganized symptoms, which have proven to have a stronger association with NC than the one they have with positive or negative symptoms (22–24).

However, when testing relationships between disorganized symptoms, NC, and MC, it is crucial to define, on the one hand, the variables (i.e., the focus on disorganized clusters or specific disorganized symptoms and the focus on fundamental vs. secondary aspects of MC), and on the other hand, the methodology for defining and assessing the variables, as it varies across different studies, which makes it difficult to compare results.

The disorganization factor [defined according to the consensus five-factor solution proposed by Wallwork et al. (25)] comprises three items of the Positive and Negative Symptom Scale (PANSS), namely, “conceptual disorganization” (CD), “difficulty in abstract thinking,” and “poor attention,” the last two presenting a possible overlap with NC impairment, whereas CD has the highest loading in the disorganization factor (26). CD consists of incoherent sequences of ideas, which results in verbosity, and atypical features such as circumstantial, illogical or tangential speech, or weakened goal of thinking and peculiar use of words and sentence constructions (27–30). Myers et al. (31) have found that only patients with formal thought disorder (FDT), as defined by PANSS CD score of ≥3, showed reduced metacognitive self-reflectivity. However, the authors did not assess NC.

A recent study from our group (32) using a network analysis to explore the relative centrality and inter-relationships between symptoms, NC, SC, MC, and real-world functioning in early and late phase SZ revealed two key findings: first, disorganized symptoms considered as a whole are a critical piece connecting NC symptoms and MC exclusively in the late-SZ group (duration of illness >5 years); second, in the whole sample, regardless of illness duration, visual learning connected NC domains with disorganization, avolition, and MC.

### 1.1 The current study

The purpose of the current study, which involves secondary data analysis from our previous study (32), was to analyze how visual learning and CD influence specific MC domains in a sample of outpatients with stable SZ.

Even though there is no unique operational definition of MC, we decided to use the Metacognitive Assessment Scale (MAS) (1), which proved to have high levels of validity and reliability and can be considered the most updated and comprehensive definition of MC (2, 4, 33, 34). Moreover, we decided to analyze not only metacognitive capacity and the total score, but also the four MC subscales: *Understanding One’s Own Mind, Understanding Others’ Mind, Decentration, and Mastery.*

Inspired by previous scientific literature, the current study has the following objectives: (1) to explore the ability of visual learning (independent variable) to predict the MC total score and the four subscales (dependent or outcome variables); (2) to explore the ability of CD to predict the outcome variables; and (3) to examine whether visual learning was able to predict the outcome variables in the presence of CD.

Given the mediating role of CD between visual learning and the outcome variables, the expectation of the study was that both visual learning and CD would interact in influencing the outcome variables.

## 2 Methods

### 2.1 Subjects

Patients with SZ according to DSM-5 criteria (35) were recruited at the Struttura Complessa Psichiatria Universitaria, Dipartimento di Neuroscienze e Salute Mentale, Azienda Ospedaliero-Universitaria “Città della Salute e della Scienza di Torino,” Turin, Italy, between January 2020 and March 2022.

#### 2.1.1 Inclusion criteria

Inclusion criteria were as follows: age between 18 and 65 years, duration of illness of ≥5 years, and SZ in a stable phase, i.e., no psychiatric hospitalization and/or treatment modifications for at least 3 months.

Two expert clinicians (CB and CM) confirmed the SZ diagnosis by means of the Structured Clinical Interview for DSM-5, Research Version (36).

#### 2.1.2 Exclusion criteria

Exclusion criteria were as follows: a current diagnosis other than SZ, substance abuse or dependence in the past 6 months, and anamnesis positive for a severe head injury (coma ≥48 h). The presence of psychiatric comorbidity and substance use disorders (SUD) was assessed using the SCID-5-TR.

#### 2.1.3 Participants

In total, 92 consecutive outpatients meeting the inclusion and exclusion criteria were recruited in the study. All patients were treated with standard care provided in community mental health centers in Italy.

All study participants provided written informed consent prior to participation.

The study complies with the Declaration of Helsinki and was conducted according to ethics committee approval (protocol number: 0057625).

### 2.2 Assessment

#### 2.2.1 Clinical assessment

The PANSS (26) was used to assess the severity of positive symptoms and disorganization. The PANSS contains 30 items rated on 1 (absent) to 7 (extreme) scales. It is designed to obtain a measure of positive (items P1–P7) and negative (items N1–N7) symptoms in patients with SZ, as well as a measure of general psychopathology (items G1–G16). We adopted the five-factor solution elaborated by Wallwork et al. (25), which comprises a positive factor (items P1, P3, P5, and G9), a negative factor (items N1, N2, N3, N4, N6, and G7), a disorganized/concrete (cognitive) factor (items P2, N5, and G11), an excited factor (items P4, P7, G8, and G14), and a depressed factor (items G2, G3, and G6), including a total of 20 items.

The Italian version of the Brief Negative Symptoms Scale (BNSS) (37) was adopted to evaluate negative symptoms. The BNSS has 13 items, organized into six subscales: anhedonia, distress, asociality, avolition, blunted affect, and alogia. For all items in the six subscales, higher scores are associated with greater impairment/presence of symptoms, with the exception of the distress item, for which the highest score is associated with the absence of negative emotions. A scale total score (ranging from 0 to 78) is calculated by summing the 13 individual items; subscale scores are calculated by summing the individual items within each subscale. The distress subscale has only one item, which quantifies the absence of distress, but this subscale is otherwise treated in the same manner as the other subscales. For the present study, we considered two factors, i.e., “avolition,” which refers to anhedonia, asociality, and experiential deficit, and “expressive deficit,” comprised of blunted affect and alogia (38).

Conceptual disorganization was assessed through an item on the PANSS (item P2) that reflects loose associations, disrupted goal-directed sequencing, and circumstantiality (39).

Depressive symptoms were evaluated using the Calgary Depression Scale for Schizophrenia (CDSS) (40).

The CDSS includes nine items (depression, hopelessness, self-depreciation, guilty ideas of reference, pathological guilt, morning depression, early wakening, suicide, and observed depression), each rated from 0 (absent) to 3 (severe). Ratings >6 on the total score indicate clinically significant depression.

#### 2.2.2 Cognitive and metacognitive assessment

The Measurement and Treatment Research to Improve Cognition in Schizophrenia (MATRICS) Consensus Cognitive Battery (MCCB) (41, 42) was used to assess NC. The MATRICS was designed to measure NC in SZ; it includes 10 subtests across seven NC domains (processing speed, attention, working memory, verbal learning, visual learning, reasoning and problem solving, and social cognition). SC, in terms of emotion processing, was evaluated using the managing emotion section of the Mayer-Salovey-Caruso Emotional Intelligence Test (MSCEIT), also included in the MCCB. The results of the MCCB were expressed as T-scores standardized for age and gender. Higher scores indicate better performance.

Metacognition was evaluated by means of the Metacognition Assessment Scale (MAS) (1), a clinician-rated scale that contains four metacognitive domains, namely, *Understanding One’s Own Mind* or *Self Reflectivity* (or the comprehension of one’s own mental states); *Understanding Others’ Mind* (or the comprehension of other individuals’ mental states); *Decentration* (or the ability to see the world as existing with others having independent motives); and *Mastery* (or the ability to use one’s mental states to foster effective action strategies in order to face cognitive tasks or cope with psychological distress) (43). The full presence of a function was assigned with a score of “1” and the partial presence of a function with a score of “0.5.” Higher scores relating to a subscale or the total scale reflect higher metacognitive abilities.

### 2.3 Procedures

Two experienced psychiatrists (CB and CM) conducted a semistructured interview to collect demographical and clinical data (age, gender, years of education, and age at illness onset) and administered PANSS, BNSS, CDSS, and MAS. To reduce inter-rater variability, first they were trained to administer according to common standards; second, at the beginning of the study, they performed independent ratings of the interviews that they conducted with the first 20 patients participating in the study. Afterward, they discussed each interview to reach consensual ratings. The agreement (within one point) between the raters varied from 80 to 95% for all PANSS items; from 80 to 90% for all BNSS items; from 85 to 95% for all CDSS items; and was 80% for the MAS total score. To maintain inter-rater reliability across the entire study period, the two raters participated every 3 months in an in-depth review of a random sample of interviews with the last author (PR).

### 2.4 Data analysis

Statistical analysis was performed using SPSS Statistics (IBM) 28.0 with a critical value of *p* of 0.05.

Mean ± standard deviation (SD) and percentages were calculated.

To test out the specific effect of visual learning on MC and the potential mediating role of CD, we analyzed the data with path-analytic techniques based on multiple regression (44).

Each path analysis was carried out in two steps: first, we tested the direct effect of visual learning on the MAS total score or their four domains; then, we tested the potential mediation of visual learning by CD (five path analytic models). In all the analyses, we statistically controlled the effects of age, schooling, gender, disease duration, positive, negative, and cognitive symptoms via multiple regression (stepwise method). To account for multiple comparisons, a Bonferroni correction was applied, and a significance level of α = 0.008 (0.05/6 = 0.008) was used for all analyses.

To test the significance of the mediation effects, we performed the Sobel test for indirect effects.

## 3 Results

Of the 92 outpatients in our sample, there were 59 male indivduals (64.1%), the mean age (mean ± SD) was 43.5 ± 10.2 years, the mean level of education (mean ± SD) was 11.2 ± 3.3 years, and the duration of illness (mean ± SD) was 20.0 ± 9.7 years. Medication protocols were as follows: unmedicated: *n* = 3 (3.2%); treatment with atypical antipsychotics: *n* = 78 (84.7%); and treatment with typical antipsychotics: *n* = 11 (11.9%). The psychopathological and cognitive characteristics of our sample are reported in Table 1.

**Table 1:** Socio-demographic, psychopathological, cognitive, functioning, and treatment characteristics of the sample.

	**(N = 92)**
Gender, *males*	59 (64.1)
Age, *years*	43.5 (10.2)
Education, *years*	11.2 (3.3)
Duration of illness, *years*	20.0 (9.7)
PANSS—Positive	9.4 (4.1)
P2	3.0 (1.5)
BNSS—Avolition	21.3 (7.9)
BNSS—Expressive deficit	14.7 (7.5)
CDSS—total score	3.7 (4.3)
MCCB—Speed of processing	24.5 (8.3)
MCCB—Working memory	28.9 (10.7)
MCCB—Reasoning and problem solving	33.6 (6.8)
MCCB—Attention	29.1 (10.7)
MCCB—Verbal learning	32.3 (8.3)
MCCB—Visual learning	35.3 (14.3)
MSCEIT—Managing emotions section	30.4 (10.6)
Treatment with atypical antipsychotics	78.0 (84.7%)
Treatment with typical antipsychotics	11.0 (12.0%)
Not in treatment with antipsychotics	3.0 (3.3%)
MAS—Total score	12.5 (6.8)
MAS—Self-reflectivity	5.4 (2.8)
MAS—Understending others’ minds	3.6 (2.1)
MAS—Mastery	2.7 (2.4)
MAS—Decentration	0.8 (1.2)

DOI, Duration of illness; PANSS, Positive and negative syndrome scale; BNSS, Brief negative symptom scale; CDSS, Calgary depression scale for schizophrenia; MCCB, Measurement and Treatment Research to Improve Cognition in Schizophrenia (MATRICS) Consensus Cognitive Battery; MSCEIT, Mayer-Salovey-Caruso Emotional Intelligence Test; MAS, Metacognition Assessment.

Control variables alone explained approximately 34.1% of variance in MAS total scores (adjusted *R*^2^ = 0.341, *p* ≤ 0.001); 32.8% of variance in MAS Self-reflectivity scores (adjusted *R*^2^ = 0.328, *p* ≤ 0.001); 30.1% of variance in MAS Understanding Others’ Mind (adjusted *R*^2^ = 0.301, *p* ≤ 0.001); 26.8% of variance in MAS Mastery (adjusted *R*^2^ = 0.268, *p* = 0.023); 10.3% of variance in MAS Decentration (adjusted *R*^2^ = 0.103, *p* = 0.066).

**Figure 1:**
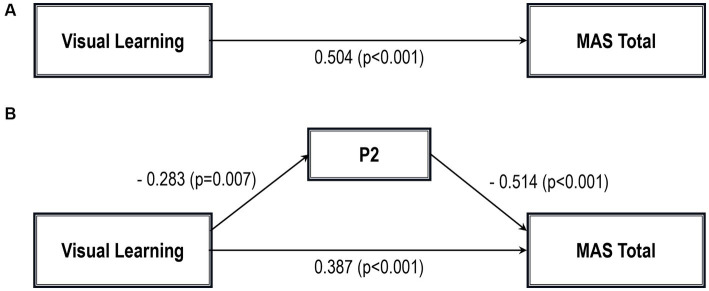
Path analysis model MAS total. **(A)** Total path. **(B)** Direct and indirect paths. MAS, Metacognition assessment scale; P2, Conceptual disorganization.

**Figure 2:**
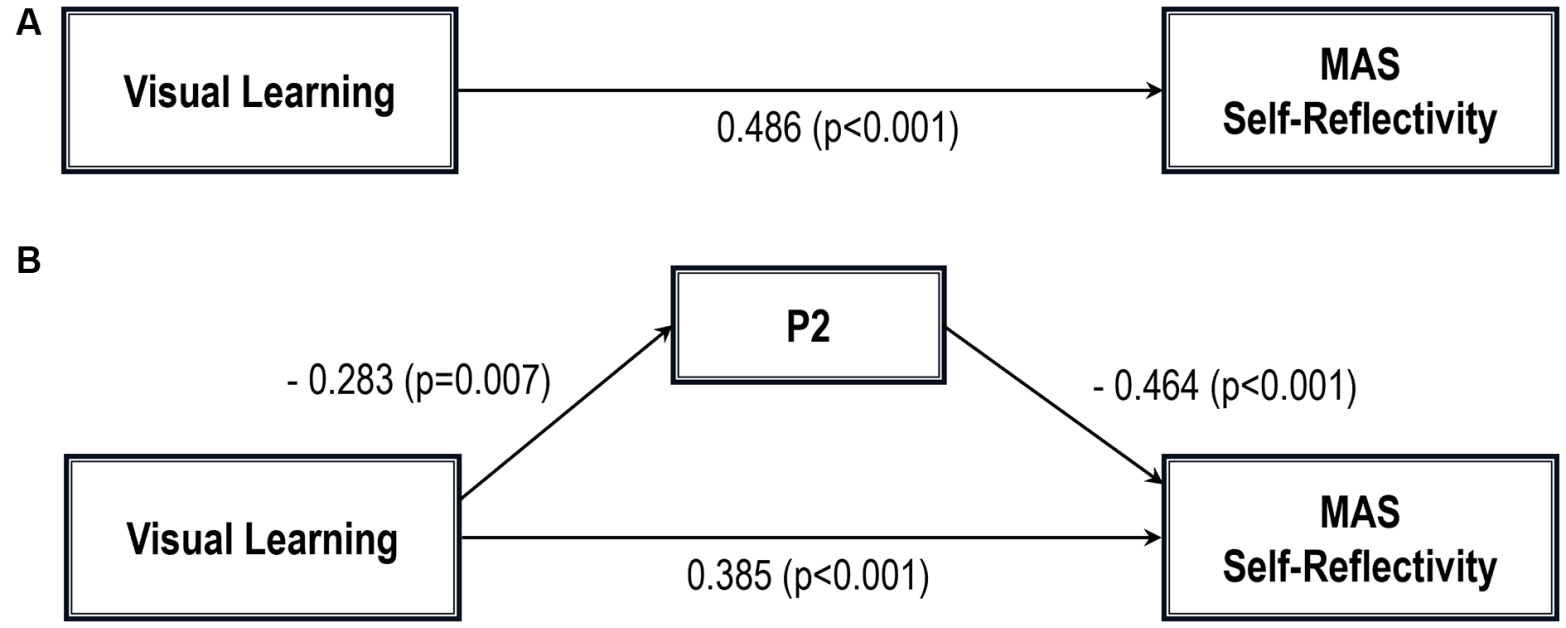
Path analysis model MAS self-reflectivity. **(A)** Total path. **(B)** Direct and indirect paths. MAS, Metacognition assessment scale; P2, Conceptual disorganization.

**Figure 3:**
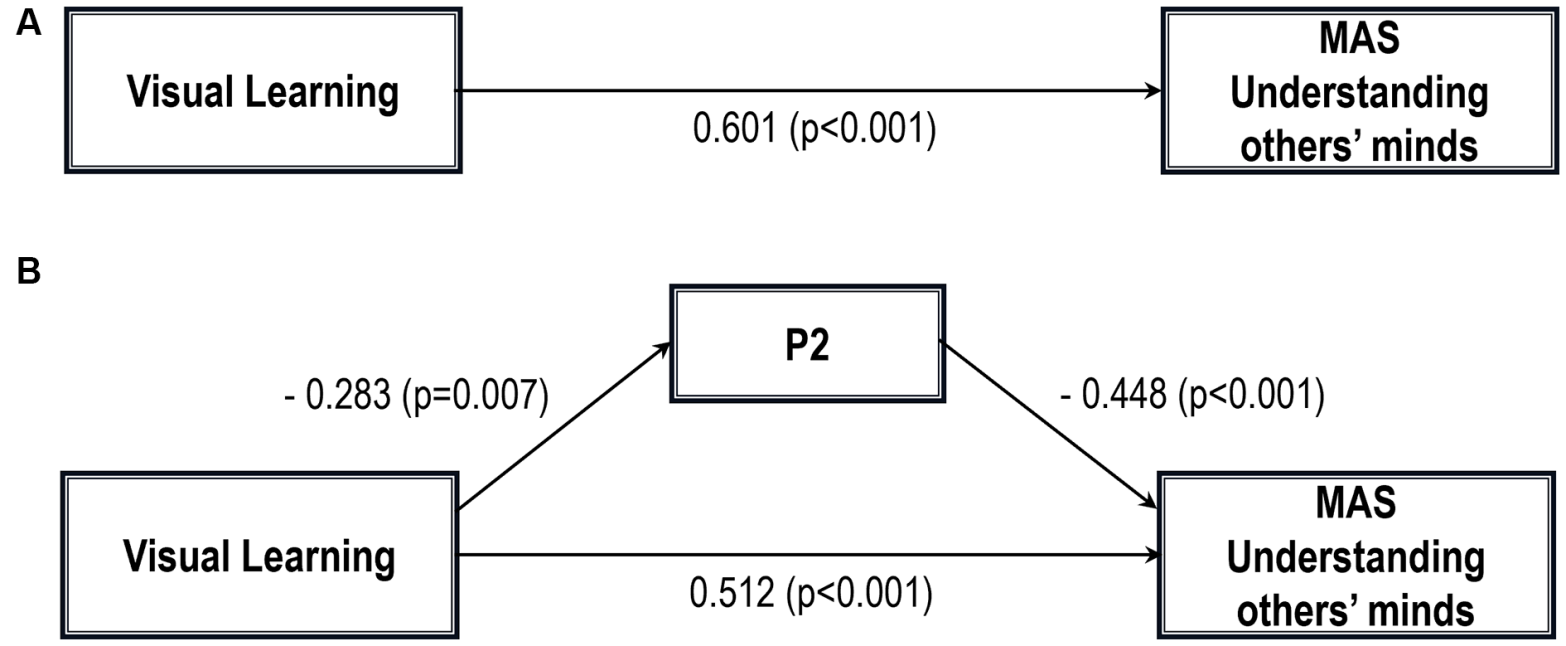
Path analysis model MAS understanding others’ minds. **(A)** Total path. **(B)** Direct and indirect paths. MAS, Metacognition assessment scale; P2, Conceptual disorganization.

**Figure 4:**
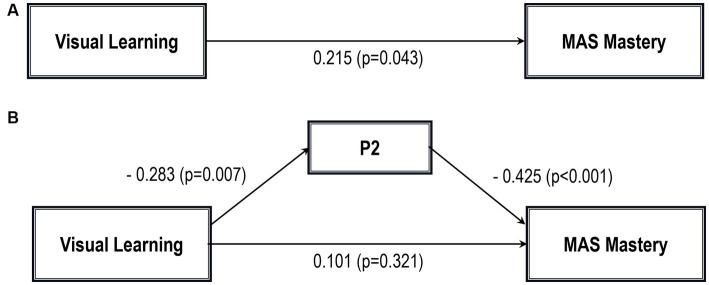
Path analysis model MAS mastery. **(A)** Total path. **(B)** Direct and indirect paths. MAS, Metacognition assessment scale; P2, Conceptual disorganization.

**Figure 5:**
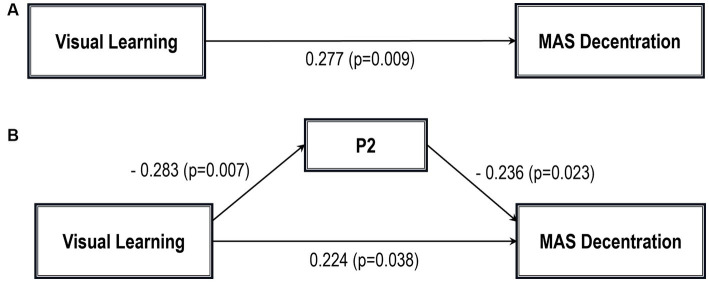
Path analysis model MAS decentration. **(A)** Total path. **(B)** Direct and indirect paths. MAS, Metacognition assessment scale; P2: Conceptual disorganization.

Five path analytic models were specified, and the path coefficients were examined. The results of path analyses are given in Tables 2–6 and Figures 1–5. The effect of visual learning was significant, showing that higher levels of visual learning predicted higher MAS total, MAS Self-reflectivity, MAS Understanding Others’ Mind, MAS Mastery, and MAS Decentration scores, after controlling for age, schooling, gender, disease duration, positive, negative and cognitive symptoms (total effect presented in Tables 2–6; see Figures 1A–5A). While there was some evidence of mediation of visual learning by differences in CD in MAS total score, MAS Self-reflectivity, MAS Understanding Others’ Mind, and MAS Decentration, the mediation was only partial. The indirect path coefficient of visual learning remained significant after inclusion of CD, while it decreased somewhat in magnitude (beta from 0.504 to 0.387 for MAS Total; beta from 0.486 to 0.385 for MAS Self-reflectivity; beta from 0.601 to 0.512 for MAS Understanding Others’ Mind; and beta from 0.277 to 0.224 for MAS Decentration; indirect path in Tables 2–4, 6; see Figures 2B, 3B, 4B, 5B).

**Table 2:** Summary of total, direct, indirect paths (standardized coefficients) MAS Total.

	beta	SE	*p*
A. Total path			
Visual learning—MAS total	0.504	0.045	<0.001
B. Indirect path			
Visual learning—MAS total	0.387	0.041	<0.001
Direct path			
Visual learning—P2	−0.283	0.011	0.007
P2—MAS total	−0.514	0.419	<0.001

MAS, Metacognition assessment scale; P2, Conceptual disorganization.

**Table 3:** Summary of total, direct, indirect paths (standardized coefficients) MAS self-reflectivity.

	beta	SE	*p*
A. Total path			
Visual learning—MAS self-reflectivity	0.486	0.018	<0.001
B. Indirect path			
Visual learning—MAS self-reflectivity	0.385	0.018	<0.001
Direct path			
Visual learning—P2	−0.283	0.011	0.007
P2—MAS self-reflectivity	−0.464	0.178	<0.001

MAS, Metacognition assessment scale; P2, Conceptual disorganization.

**Table 4:** Summary of total, direct, indirect paths (standardized coefficients) MAS understanding others’ minds.

	beta	SE	*p*
A. Total path			
Visual learning—MAS understanding others’ minds	0.601	0.013	0.001
B. Indirect path			
Visual learning—MAS understanding others’ minds	0.512	0.012	0.001
Direct path			
Visual learning—P2	−0.283	0.011	0.007
P2—MAS understanding others’ minds	−0.448	0.122	<0.001

MAS, Metacognition assessment scale; P2, Conceptual disorganization.

**Table 5:** Summary of total, direct, indirect paths (standardized coefficients) MAS mastery.

	beta	SE	*p*
A. Total path			
Visual learning—MAS mastery	0.215	0.017	0.043
B. Indirect path			
Visual learning—MAS mastery	0.101	0.017	0.321
Direct path			
Visual learning—P2	−0.283	0.011	0.007
P2—MAS mastery	−0.425	0.153	<0.001

MAS, Metacognition assessment scale; P2, Conceptual disorganization.

**Table 6:** Summary of total, direct, indirect paths (standardized coefficients) MAS decentration.

	beta	SE	*p*
A. Total path			
Visual learning—MAS decentration	0.277	0.009	0.009
B. Indirect path			
Visual learning—MAS decentration	0.224	0.009	0.038
Direct path			
Visual learning—P2	−0.283	0.011	0.007
P2—MAS decentration	−0.236	0.082	0.023

MAS, Metacognition assessment scale; P2, Conceptual disorganization.

However, when the mediation effect of CD was taken into account, visual learning was no longer a significant predictor of MAS Mastery on its own, although the sign of the coefficient remained the same (indirect effect in Table 5; see also Figure 5B).

In addition, Sobel tests for mediation showed that CD significantly mediated the relationship between visual learning and MAS Self-reflectivity (*Z* = 1.83; *p* = 0.03); MAS Understanding Others’ Mind (*Z* = 2.04; *p* = 0.02); MAS Mastery (*Z* = 1.88; *p* = 0.02); and MAS Decentration (*Z* = 1.91; *p* = 0.02). The Sobel test for the MAS total score did not reach statistical significance.

## 4 Discussion

The purpose of the present study was to investigate the relationship between visual learning and CD in predicting MC within a demographic sample of middle-aged outpatients in late-phase SZ who are in a stable phase of their illness. Three key findings emerged.

First, as expected, it was discovered that outpatients with greater levels of visual learning also had stronger metacognitive capacities, including Self-reflectivity, Understanding Others’ Mind, Decentration, and Mastery.

Even if it has been hypothesized that MC, symptoms, and NC influence one another bidirectionally (23, 45, 46), visual learning has been found to predict conversion to psychosis among clinical high-risk (CHR) patients (47, 48) and has been shown to be more central than other NC domains in network models investigating the relationships between psychopathology, NC, MC, and real-world functioning in SZ (7, 25). Indeed, given that visual learning measures the ability to locate and remember things in space, it could affect individuals’ ability to think about themselves and others and to understand how events are influenced by one another. This could compromise the ability to assess the accuracy of our internal perceptual state and the integrated sense of our perceptual environment, that depends on whether we can predict upcoming sensory information in integrative manner. Thus MC representing a postperceptual decision-making process (7, 49). This is in line with the hypothesis that impaired formation of visual percepts can lead to problems in higher-level processing and with theoretically based models of pathways to functional outcome in SZ starting from microlevel early visual perception (50).

Second, as for CD, our study yielded two main outcomes: first, we found a negative association between visual learning and CD; second, greater severity of CD was negatively associated with increasing levels of MC abilities. It is hard to make comparisons among studies because earlier works analyzed mostly disorganized symptoms instead of CD, a crucial item in the definition of disorganization. We chose to focus on CD because it has been correlated more than other aspects with NC dysfunction (51) and because it resembles Bleuler’s concept of “loosening of associations,” i.e., the central mechanism underlying disturbances in thinking, motivation, and affective expression. However, our results obviously replicate the findings of the previous study of our group (32), which found that disorganization and visual learning not only exhibited high centrality indices, but also seemed to be consistent with a meta-analysis (23) reporting that disorganization was associated with all NC domains. Generally, individuals with NC impairments express a more disorganized speech, making it difficult for listeners to discern the essential information needed to bind the speaker’s ideas.

As for the relationship between CD and MC and their subscales, we found that, as thinking becomes more disordered and less goal-directed, patients display a reduced ability to think about their own thinking or engage in self-reflective processes, i.e., MC decreases. Our results are consistent with the findings of the meta-analysis by Arnon-Ribenfield (21), which reported strong negative associations between MAS subscales and PANSS factors. Following that meta-analysis, 18 further studies (52–69) have been published: the sample size ranges from 6 (66) to 324 patients (63); only four studies included NC measures (57, 58, 61, 68); and most of them adopted a selection of different MC scales, capturing different MC types and aspects that could have differential relationships with each of the different disorganized symptoms.

However, when we analyze research literature that focuses on a single item of disorganization instead of all symptoms, the effect size depends on the level of the symptoms in the sample, i.e., becoming higher at higher levels of disorganized speech (20, 31).

Of course, a number of hypotheses have been proposed concerning the strong association between MC capacity and disorganized symptoms. First, it has been suggested that disorganized symptoms and MC capacity share conceptual links. Individuals who find it difficult to organize their ideas, concepts, and feelings coherently would also exhibit difficulties in the integration of internal experiences (i.e., thoughts and feelings) together in a cohesive framework. Second, the presence of a correlation between these variables does not automatically assume a causal relationship between them; anyway, the fact that significant disorganized symptoms, when present, may impact an individual’s MC capacity is a possibility to be considered. Third, the strong association between the two constructs could be due to the selection of psychometric scales, consequently artificially amped up.

Third, when CD was included in the five models as a mediating variable between visual learning and MC and its scales, the effect of the former on the latter’s kept the positive sign, even though the effect was reduced.

A variable can be viewed as a mediator (DC) insofar as it takes into account the relationship between a given independent variable (IV) (visual learning) and a given dependent variable(s) (DV) (MAS total and its four scales) (70). As stated by Baron and Kenny (70) and Judd and Kenny (71), partial mediation can occur after controlling for the mediator when the IV effect on DV decreases by a non-trivial amount but not to zero, as it happens for MAS Self-reflectivity, MAS Understanding Others’ Mind, and MAS Decentration. The perfect mediation occurs when the direct effect is no longer significant after considering the mediator, as for MAS Mastery in our article. Indeed, the relationship between visual learning and MAS Mastery can be completely explained by their relationships with CD.

The above-mentioned results partially replicate those (20, 31) that have shown that CD modulates the moderating effect of disorganized symptoms on the relationship between NC and MC.

Overall, the finding that CD mediates the relationship between NC and specific MC types shows that the term “metacognition” includes a wide range of processes rather than a single construct, each of them describing different aspects of MC. For example, in our study, only Mastery, a domain of MC that measures the capacity to use the understanding of mental states to face psychological challenges, was no longer explained by NC when CD was taken into account.

### 4.1 Limitations and strengths

Some limitations should be considered in the interpretation of our results. First, the sample size of the present study was relatively small, even if it was in line with previous studies. Second, we enrolled mainly middle-aged outpatients engaged in treatment and in a stable phase of their disorder. Thus, our results cannot be generalized to other populations, i.e., inpatients or patients in more acute phases of their illness, or those who are drug-naïve or those who refuse treatment. Third, the cross-sectional design did not allow us to establish a cause-and-effect relationship. Thus, future longitudinal studies are needed to investigate the directionality of our findings as well as to identify other variables that may influence these relationships. Fourth, we measured CD only using one clinician-rated item obtained using the PANSS, and no behavior-based measures of disorganized speech were used. These measures would allow to identify disorganization in speech samples using either trained raters or automated analysis. Fifth, even though MAS-A has been considered an established tool to evaluate the four sub-dimensions of synthetic MC, a recent psychometrical analysis (55) on 130 outpatients with a diagnosis of SZ or schizoaffective disorders has shown that the latent structure of the MAS-A might be essentially one-dimensional.

Notwithstanding these limitations, our study has some strengths.

First, path analyses allowed us to investigate the relationships among the identified variables and estimate the magnitude and hypothesized causal connections between sets of variables. Second, we assessed NC, the “third” variable (31) often omitted in studies analyzing the relationship between MC and disorganization.

## 5 Conclusion and implications

If replicated, findings from this study could inspire interventions designed to improve MC in patients with stable SZ, i.e., targeting NC or targeting CD. Indeed, we think that if CD and visual learning underlie MC, then our findings may have implications for treatments that address CD and NC. Indeed, these interventions could have an impact on MC; that is, visual learning and CD improvement could help in attributing meaning to experiences and integrating them into larger mental representations of self, others, and the world. However, studies on interventions that target CD or NC rarely include tools to evaluate MC as an outcome.

Moreover, when interpreting our data, it is important to consider two further topics. First, there is no evidence for simple direct relationships among NC, disorganization, and MC, as yet unidentified variables or mediators could intervene in this relationship. Second, results provide further evidence that MC represents a wide spectrum of processes (14): MC domains are indeed separate capacities, such that each one may be influenced by different variables. Therefore, studies unable to distinguish between different components of MC fail to bring out the possibly varying links between NC, disorganization, and MC.

## Data availability statement

The datasets presented in this article are not readily available because the full datasets contain identifying information, and data sharing is subject to facility guidelines. Requests to access the datasets should be directed to the CM and paola.rocca@unito.it.

## Ethics statement

The studies involving humans were approved by Research Ethics Committee AOU Città della Salute e della Scienza di Torino. The studies were conducted in accordance with the local legislation and institutional requirements. The participants provided their written informed consent to participate in this study. Written informed consent was obtained from the individual(s) for the publication of any potentially identifiable images or data included in this article.

## Author contributions

CM: Conceptualization, Data curation, Investigation, Methodology, Supervision, Writing – original draft, Writing – review & editing. CB: Conceptualization, Methodology, Writing – review & editing. SB: Investigation, Methodology, Supervision, Writing – review & editing. PB: Conceptualization, Methodology, Supervision, Writing – review & editing. VV: Conceptualization, Methodology, Supervision, Investigation, Writing – review & editing. PR: Conceptualization, Supervision, Methodology, Writing – review & editing.
